# Multi-Terminal Spin Valve on Channels with Spin-Momentum Locking

**DOI:** 10.1038/srep35658

**Published:** 2016-10-21

**Authors:** Shehrin Sayed, Seokmin Hong, Supriyo Datta

**Affiliations:** 1 School of Electrical and Computer Engineering, Purdue University, West Lafayette, IN 47907, USA

## Abstract

It is experimentally established that charge current flowing in a channel with spin-momentum locking such as topological insulator surface states or Rashba interfaces induces a spin voltage, which can be electrically measured with a ferromagnetic contact along the current path. Using this fact in conjunction with Onsager reciprocity arguments, we make the surprising prediction that the anti-parallel resistance of a spin valve can be either larger or smaller than the parallel resistance depending on the direction of spin flow relative to the direction of spin-momentum locking. However, we argue that this remarkable signature of spin-momentum locking can only be observed in multi-terminal measurements. Two-terminal measurements in the linear response regime, will show a single anti-parallel resistance larger than the parallel resistance as commonly observed in channels without spin-orbit coupling. We support this result with detailed numerical calculations based on a semiclassical model that provides insight into the underlying physics.

Topological spintronics is a topic of great current interest especially because of the possibility of efficient spin-charge conversion not only in topological insulators (TI) (see, for example, refs 1, 2, 3, and references therein) but in 2D conductors with large spin-momentum locking (SML) in general. It now seems clearly established that a charge current flowing in 2D conductors with SML creates a spin voltage that can be measured with ferromagnetic (FM) contact(s) in a multi-terminal setup. For example, if we measure the change in voltage at one of the FM contacts due to a change in its magnetization (say *m*_1_) in Fig. 1(a), it is proportional to the current *I*_34_ flowing between the non-magnetic (NM) contacts. This was originally observed^4^,^5^ and analyzed^6^ in semiconductors like InAs. Later, it was shown^7^,^8^ that this spin voltage should be observed in TI surface states as well, and both phenomena could be understood in terms of a single parameter which we called the channel polarization *p*_*c*_, given by


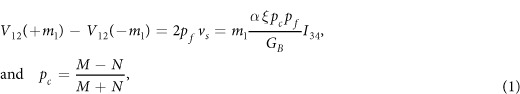


where *M*, *N* represent the number of modes for forward-moving up-spin and down-spin states, which are also equal for backward-moving down-spin and up-spin states respectively due to time-reversal symmetry (TRS). *M* and *N* are evaluated at the Fermi energy for zero temperature and in general require thermal averaging. *α* = 2/*π* is an angular averaging factor for the 2D channel^7^,^8^ and *p*_*f*_ is the polarization of the FM contacts, which is assumed to be the same for both. The factor *ξ* depends on the details of the structure and can be evaluated from our detailed semiclassical model presented in a later section for a specific structure. For high resistance contacts *ξ* is usually ~1, but can be significantly less than 1 for low contact resistance. *G*_*B*_ = (*q*^2^/*h*)(*M* + *N*) is the ballistic conductance related to the total number of modes (*M* + *N*) in the channel which represents a material property of the channel and does not imply ballistic transport. The results discussed in this paper are valid all the way from the ballistic to the diffusive regime.

The polarization of Rashba interfaces with a coupling coefficient *α*_*R*_ is approximately given by^6^,^7^ *p*_*c*_ ~ *α*_*R*_*k*_*F*_/(2*E*_*F*_) which is usually much less than one, *k*_*F*_ and *E*_*F*_ being the Fermi wave number and Fermi energy respectively. In principle, for a perfect TI the polarization *p*_*c*_ ~ 1 (i.e. *N* = 0). However, in reality there are parallel channels which dilute the effective *p*_*c*_. The spin voltage predicted in ref. 7 has been confirmed experimentally with potentiometric measurements on TI by a number of experimental groups^9^,^10^,^11^,^12^,^13^,^14^,^15^,^16^.

Similar measurements have been reported on gold^17^ and could be observable on other heavy metals such as platinum^18^,^19^, tantalum^20^,^21^, tungsten^22^. The underlying mechanism of the spin polarization in these metals are still being debated^23^,^24^,^25^ and could involve either a bulk spin Hall effect^21^,^26^,^27^ or interface Rashba-like channel^28^,^29^,^30^,^31^,^32^. Irrespective of the mechanism, materials as diverse as semiconductors (like InAs), topological insulators (like Bi_2_Te_3_) or heavy metals (like gold) show similar terminal characteristics^25^,^33^ and as long as the underlying mechanism is surface-related, Eq. (1) relates *V*_12_/*I*_34_ to *p*_*c*_ describing a key property of the surface channel. Although the quantitative model we present is based on such surface channels, the predictions in this paper follow rigorously from the principle of reciprocity and should be of interest in any material that exhibits a non-zero change in the voltage *V*_12_ upon magnetization switching in the presence of a current *I*_34_, irrespective of the underlying mechanism.

In this paper, we wish to draw attention to a surprising prediction that follows from this well-established potentiometric result simply by invoking Onsager reciprocity relation^6^,^34^,^35^. Specifically, we will show in in the next section that for the setup in Fig. 1(a), the four-terminal (4T) resistance is given by


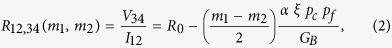


where *V*_34_ is the measured voltage between two non-magnetic (NM) contacts 3 and 4, *I*_12_ is the supply current between two ferromagnetic contacts 1 and 2 whose magnetization along 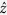 are given by *m*_1_ and *m*_2_ respectively. *m*_1,2_ = +1 or −1 corresponds to magnetization along 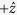 or 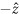 respectively. *R*_0_ is a magnetization-independent resistance that depends on the spatial separation between the FM contacts. Note that it follows from Eq. (2) that


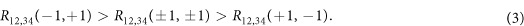


This means that if we perform an experiment using two FM contacts of different coercivity and sweep an external magnetic field (*B*_*ext*_) in both directions, we should see a variation like Fig. 1(b) as the magnets *m*_1_ and *m*_2_ switch at different magnetic fields creating different (*m*_1_, *m*_2_) combinations. This is the standard experiment commonly done on two-terminal (2T) spin valves and usually leads to the result shown in Fig. 1(c) with an anti-parallel resistance *R*_*AP*_ = *R*_12,12_(+1, −1) = *R*_12,12_(−1, +1) that is greater than the parallel resistance *R*_*P*_ = *R*_12,12_(+1, +1) = *R*_12,12_(−1, −1). In channels with SML described by the channel polarization *p*_*c*_, however, we are predicting a very different result as shown in Fig. 1(b), with two separate anti-parallel 4T resistances (see Eq. (3)), depending on the direction of spin flow relative to the spin-momentum locking.

Note that this remarkable signature of spin-momentum locking can only be observed in multi-terminal measurements. A similar result has been proposed^36^ for a 2T structure on topological insulator. However, we argue from Onsager reciprocity relation that 2T measurements in the linear response regime will only show the usual result^37^,^38^,^39^,^40^,^41^ (see Fig. 1(c))


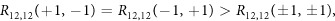


even in channels with spin-momentum locking. To our knowledge, there is no experimental evidence of three distinct states on 2T TI spin valve like structure^42^.

Equation (2) is the central result of this paper which we first establish using simple reciprocity arguments in the next section, followed by a detailed semiclassical model, which is used to provide insight into the physics behind Eqs (2) and (3).

## Simple Justification

### Potentiometric measurement

A charge current flowing in an arbitrary channel causes a separation between electrochemical potentials for forward (*μ*^+^) and backward (*μ*^−^) moving states^43^. With spin-orbit coupling present in the channel, this separation is reflected in a separation in the electrochemical potentials for up (*μ*_*up*_) and down (*μ*_*dn*_) spin polarized electrons due to spin-momentum locking. Indeed as we will discuss in a later section that we can write *μ*_*up*_ − *μ*_*dn*_ ≈ *αp*_*c*_(*μ*^+^ − *μ*^−^) under the assumption that reflection with spin-flip is the dominant scattering mechanism in the channel. The resulting spin voltage *v*_*s*_ = (*μ*_*up*_ − *μ*_*dn*_)/(2*q*) (*q*: electron charge) can be measured with a FM contact^7^ along the current path (see Fig. 2(a)), given by Eq. (1).

For a setup with two FM contacts (see Fig. 2(b)), *V*_12_ registers a voltage that depends on the difference in the magnetizations of the two contacts, along with an offset voltage (*V*_*os*_) which depends on the spatial separation of the two FM contacts


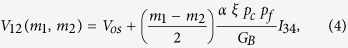


where *p*_*f*_ is the polarization of both FM contacts.

### Reciprocal measurement

Potentiometric measurement on a material described by time-reversal invariant (TRI) Hamiltonian should satisfy Onsager reciprocity relation^6^,^34^,^35^: *R*_*ij*,*kl*_(+*m*) = *R*_*kl*,*ij*_(−*m*), where *R* is the resistance, first and second pair of indices denote supply current terminals and measured voltage terminals respectively. Thus reciprocity requires that


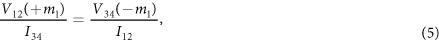


which combined with Eq. (1) yields the measured voltage of the setup in Fig. 2(c), given by


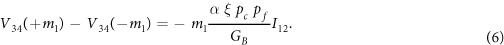


The setup in Fig. 2(c) is reciprocal to the setup in Fig. 2(a). Note the negative sign arising from the reversal of the magnetization which is required to satisfy the reciprocity relation. The reciprocal relation between Eqs (1) and (6) including the negative sign has been demonstrated experimentally by Liu *et al*.^10^. Moreover, we believe that the three terminal experimental measurement on TI by Ando *et al*.^44^ can be interpreted with the effect in Eq. (6), although Ando *et al*. interpreted their results in terms of an effect similar to that in Eq. (1), but based on a proposal of 2T setup^45^. We believe that in order to observe effects in Eqs (1) and (6), it is important to use a multi-terminal measurement in the linear response regime.

The Onsager reciprocity relation for a setup with two FM contacts is given by


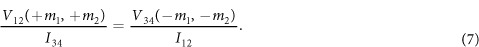


**Figure 1:**
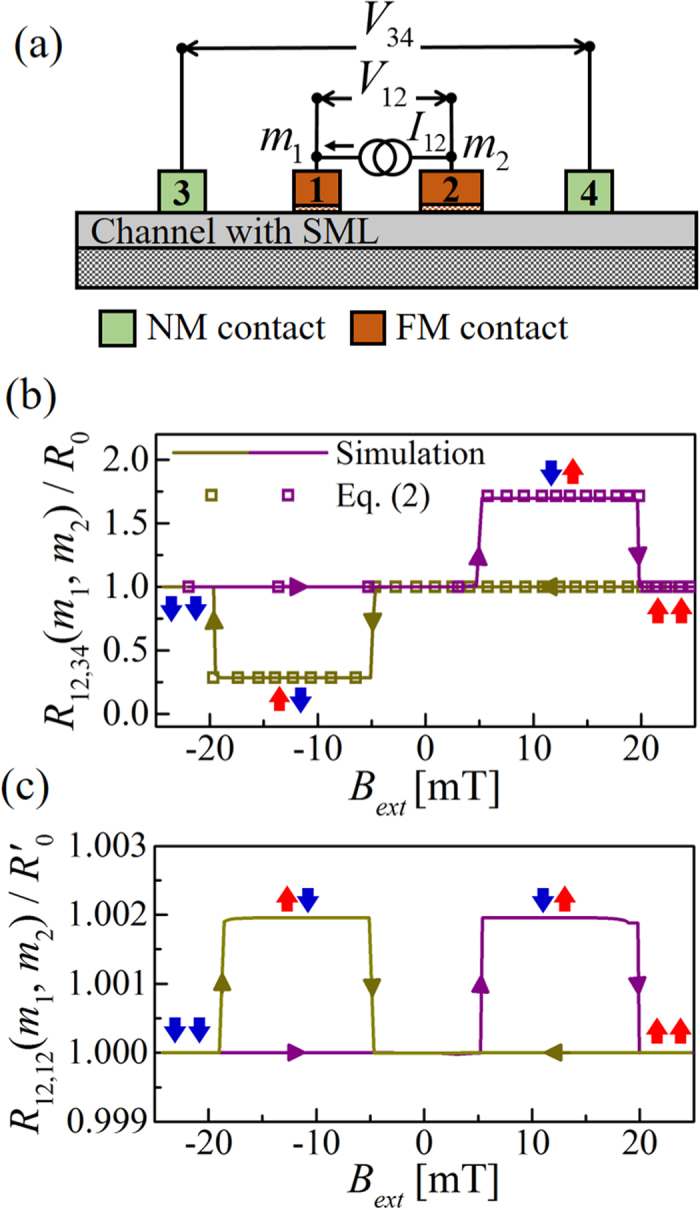
(**a**) Four-terminal (4T) spin valve like setup on channel with spin-momentum locking (SML). Supply current *I*_12_ running between ferromagnetic (FM) contacts 1 and 2 and voltage *V*_34_ is measured between non-magnetic (NM) contacts 3 and 4. (**b**) *R*_12,34_ = *V*_34_/*I*_12_ as a function of an external magnetic field (*B*_*ext*_). We observe two separate anti-parallel resistance such that *R*_12,34_(−1, +1) > *R*_12,34_(±1, ±1) > *R*_12,34_(+1, −1), depending on the direction of spin flow relative to the SML. Simulation was compared to Eq. (2) for *p*_*c*_ = 0.8, *p*_*f*_ = 0.9, and *ξ* ≈ 1. (**c**) *R*_12,12_ = *V*_12_/*I*_12_ as a function of *B*_*ext*_ show the usual result *R*_12,12_(+1, −1) = *R*_12,12_(−1, +1) > *R*_12,12_(±1, ±1) even in channels with SML. *B*_*ext*_ is swept in both direction between −25 mT to +25 mT as indicated by the arrows in the curves. Coercive fields of the FM contacts 1 and 2 are 20 and 5 mT, respectively. Red up and blue down arrows indicate magnetization direction *m*_1,2_ = +1 and −1 respectively. Note that resistances in the plot are normalized to the offset resistance. Simulation was performed with a detailed semiclassical model (see Eq. (11)), with parameters: *λ* ≈ 100 nm and total number of modes ≈100. The contacts 1–4 are spaced by 2*λ*.

**Figure 2:**
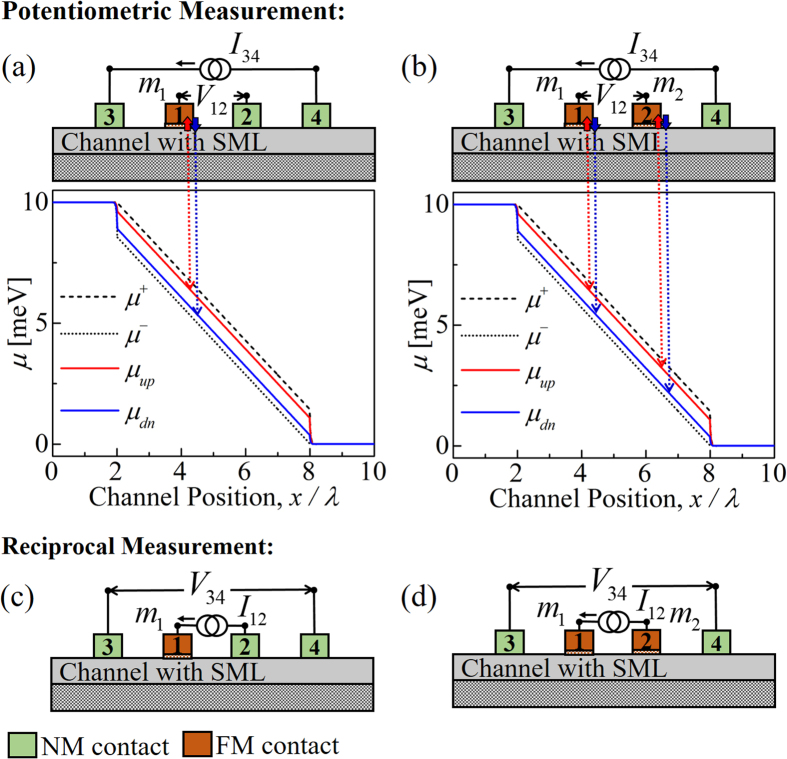
Potentiometric measurement. Charge current (*I*_34_) in an arbitrary channel causes a separation between electrochemical potentials for forward (*μ*^+^) and backward (*μ*^−^) moving states. Spin-orbit coupling present in the channel induces a spin voltage *qv*_*s*_ = *μ*_*up*_ − *μ*_*dn*_ ≈ *αp*_*c*_(*μ*^+^ − *μ*^−^) which can be measured with ferromagnetic (FM) contact(s). The spin voltage is the (**a**) change in *V*_12_ in one FM setup upon *m*_1_ switching (Eq. (1)) or (**b**) only *V*_12_ in two FM setup for (*m*_1_, *m*_2_) = (+1, −1) or (−1, +1). Different (*m*_1_, *m*_2_) gives three distinct measurements given by Eq. (4). *V*_*os*_ is the offset voltage which depends on the spatial separation between two FM contacts. **Reciprocal measurement:** Onsager reciprocity relation yields measurements reciprocal to the (**c**) setup in (**a**) which corresponds to Eq. (6) and (**d**) setup in (**b**) which corresponds to Eq. (2). This setup is same as that in Fig. 1(a). Note that channel position is normalized to the mean free path (*λ*). Parameters: *p*_*c*_ = 0.5, *λ* = 100 nm, and total number of modes ≈100.

The reciprocal setup of Fig. 2(b) is shown in Fig. 2(d) which was introduced as the 4T spin valve like setup in Fig. 1(a). The central result in Eq. (2) can be derived by combining Eq. (4) with Eq. (7), which gives the three states of 4T resistance *R*_12,34_(*m*_1_, *m*_2_) = *V*_34_(*m*_1_, *m*_2_)/*I*_12_. The offset resistance is given by *R*_0_ = *V*_*os*_/*I*_12_. Note that this unique signature of spin-momentum locking in the channel should not be observed in 2T linear response measurements, because reciprocity requires that *R*_12,12_(+*m*_1_, +*m*_2_) = *R*_12,12_(−*m*_1_, −*m*_2_).

### Inverse Rashba-Edelstein Effect

The setup in Fig. 2(d) can be used to quantify the spin pumping induced charge voltage in the channel, often known as the inverse Rashba-Edelstein effect (IREE)^46^,^47^,^48^, if spin pumping is visualized as electrical injection with two perfect half metallic FM contacts (i.e. *p*_*f*_ ≈ 1) having opposite magnetization directions (e.g. (*m*_1_, *m*_2_) = (+1, −1)). These conceptual contacts are located at the same point in space so that the offset resistance *R*_0_ is zero. The charge current running between FM contacts 1 and 2 injects a pure spin current (*i*_*s*_) in the channel (i.e. *I*_12_ = −*i*_*s*_/2 since up spins are flowing into the channel from contact 1 and down spins are flowing out of the channel from contact 2). The open circuit charge voltage across NM contacts 3 and 4 is given by 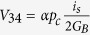 from Eq. (2). The short circuit current (*I*_*sc*_) is related to the open circuit voltage through *I*_*sc*_ = *GV*_34_, *G* being the channel conductivity *G*_*B*_*λ*/*L*, *λ* and *L* are the mean free path and length of the channel respectively. This leads to a simple expression for the IREE length^46^,^47^,^48^ as


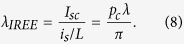


Note that this expression agrees with the result of ref. 46, noting that for weak Rashba coupling the channel polarization *p*_*c*_ = *α*_*R*_*k*_*F*_/(2*E*_*F*_).

## Semiclassical Model

### Four electrochemical potentials

**Figure 3:**
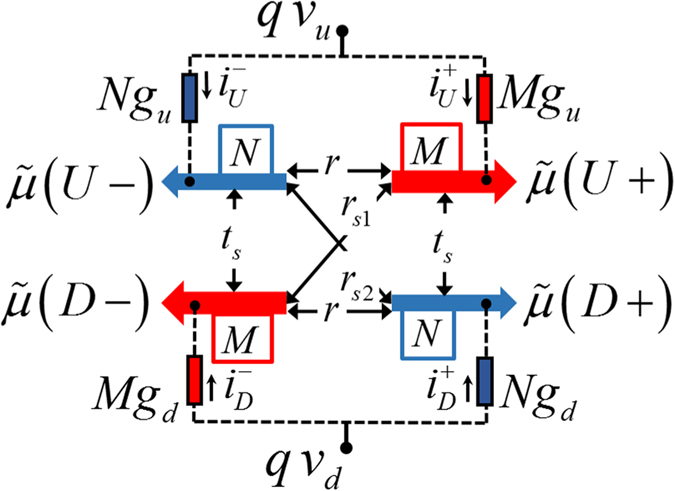
Channel electronic states are classified into four groups of states based on the spin polarization (up (*U*), down (*D*)) and the sign of the group velocity (+, −). Electrochemical potentials for these groups are: 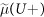, 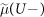, 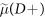, and 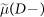. Spin-orbit coupling requires different number of modes for up and down spin states and time reversal symmetry requires that (*U*+, *D*−) pair have same number of modes *M* and (*U*−, *D*+) pair have same number of modes *N*. We consider three types of scattering mechanisms: reflection with (*r*_*s*1,2_) and without (*r*) spin-flip and transmission with spin-flip (*t*_*s*_). *r*_*s*1,2_, *r*, and *t*_*s*_ are scattering rates per unit length. External excitation is modeled as up (*v*_*u*_) and down (*v*_*d*_) spin voltages applied to up and down states of the channel through up and down spin conductances per mode per unit length (*g*_*u*_ and *g*_*d*_), respectively. Four different currents (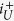, 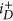, 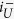, and 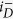) enter the four different groups in the channel. The contact is a non-magnetic (NM) contact if *g*_*u*_ = *g*_*d*_ or a ferromagnetic (FM) contact if *g*_*u*_ ≠ *g*_*d*_.

We have extended our previously reported semiclassical model^8^ for 2D channels with spin-momentum locking (SML) (e.g. topological insulator surface states, Rashba interface etc.) to include the effect of different contacts: (a) non-magnetic contact(s) and (b) ferromagnetic contact(s). The model uses four electrochemical potentials (see Fig. 3) by classifying all the electronic states in the channel into four groups based on the spin polarization (up (*U*) or down (*D*)) and the sign of the group velocity (+ or −). Spin-orbit coupling in the channel requires that the number of modes for up and down spins to be different (e.g. *U*+ and *D*+ have *M* and *N* number of modes respectively) and time-reversal symmetry requires that the number of modes for up spins going forward (*U*+) or backward (*U*−) be the same as the number of modes for down spins going backward (*D*−) or forward (*D*+) respectively. *M*, *N* represent the thermally averaged number of modes as stated earlier (see Eq. (1)). The electrochemical potentials for forward and backward moving states in the channel are given by


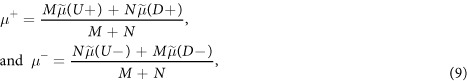


and the electrochemical potentials for up and down spin polarized states in the channel are given by


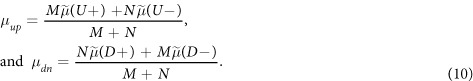


Note that for SML channel with polarization *p*_*c*_ ~ 1 (i.e. *N* ≈ 0) we have 
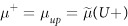
 and 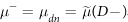.

### Generalized spin diffusion equation

We consider three types of scattering mechanisms in the channel: reflection with spin-flip (*r*_*s*1,2_), reflection without spin-flip (*r*), and transmission with spin-flip (*t*_*s*_) *r*_*s*1,2_, *r*, and *t*_s_ are scattering rates per unit length. The following 1D differential equation describes a channel with SML


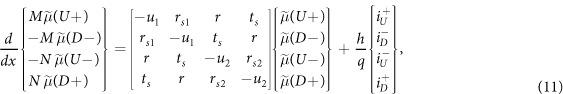


where *u*_1_ = *r*_*s*1_ + *r* + *t*_*s*_ and *u*_2_ = *r*_*s*2_ + *r* + *t*_*s*_. *h* is the Planck constant. Note that the electrochemical potentials in this equation are subtracted from their equilibrium state i.e. 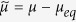. This model partially appeared in our previous work^8^ with additional term taking into account the four different currents going into the four groups in the channel from the external contact. The model does not account for effects such as spin precession, those involves off-diagonal elements of the density matrix which we assume to be negligible.

### Contact currents

The effect of external contact is modeled as external up and down spin voltages (*v*_*u*_ and *v*_*d*_) being applied to up (*U*+, *U*−) and down (*D*+, *D*−) spin polarized channel states through up and down spin conductances per mode per unit length (*g*_*u*_ and *g*_*d*_), respectively. The four currents from the external contact are given by


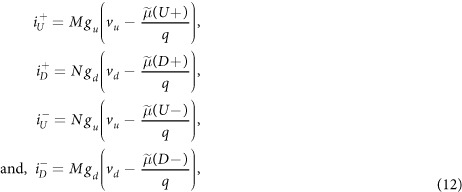


where the contact can be a FM or NM given that *g*_*u*_ ≠ *g*_*d*_ or *g*_*u*_ = *g*_*d*_ respectively. The currents at the two ends of the 1D channel are given by


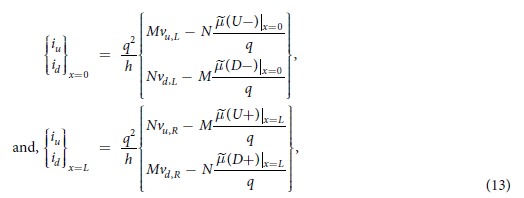


which can be treated as contacts with spin-orbit coupling. Here, 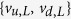 and 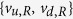 are up/down spin voltages applied at left and right boundaries of the 1D channel with SML and *L* is the channel length. We numerically solve Eq. (11) to observe electrochemical potential profile along the channel and calculate the contact spin and charge currents/voltages using Eqs (12) and (13). Details of the numerical analysis are provided as supplementary information.

## Results and Discussion

### Parameters

**Figure 4:**
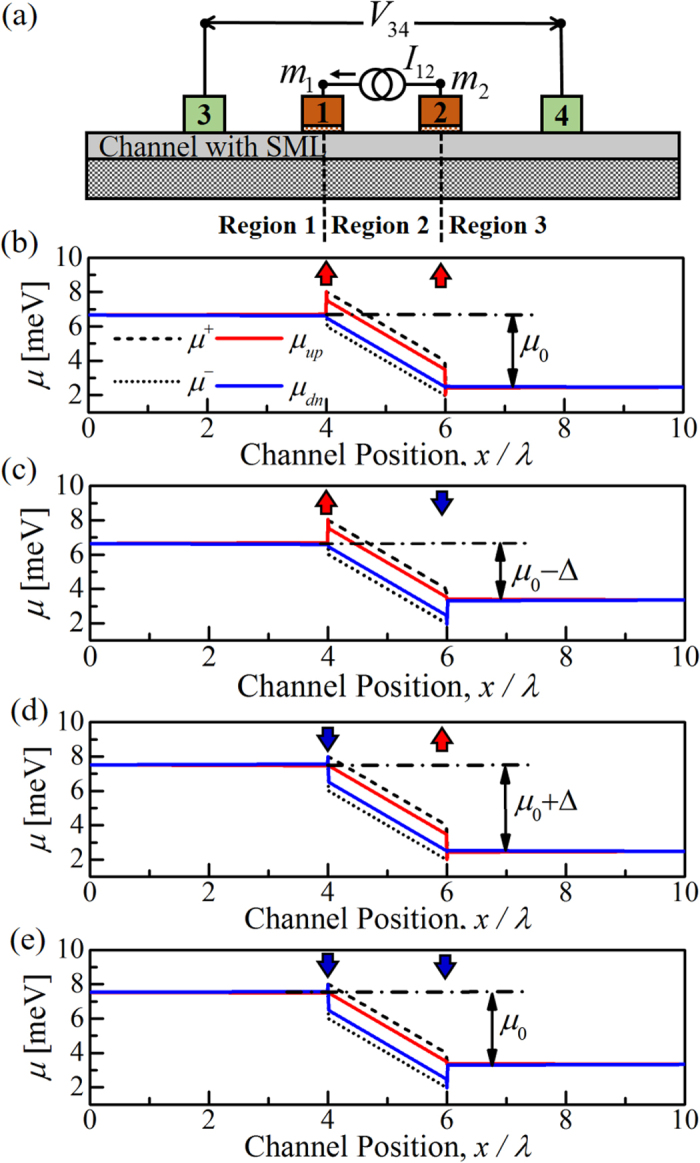
Simulation of the setup in Fig. 1(a) using Eq. (11). We observe electrochemical potentials (Eqs (9) and (10)) along the channel for different FM contact magnetizations (*m*_1_, *m*_2_): (**a**) (+1, +1), (**b**) (+1, −1), (**c**) (−1, +1), and (**d**) (−1, −1). Red up and blue down arrows indicate *m*_1,2_ = +1 and −1 respectively. *I*_12_ causes a separation between *μ*^+^ and *μ*^−^ in region 2 and spin-orbit coupling in the channel creates a spin potential *μ*_*up*_ − *μ*_*dn*_ ≈ *αp*_*c*_(*μ*^+^ − *μ*^−^). No separation between electrochemical potentials in regions 1 and 3 as no charge current is flowing. The difference between average electrochemical potentials in regions 1 and 3 is minimum (*μ*_0_ − Δ) for (+1, −1) configuration and maximum (*μ*_0_ + Δ) for (−1, +1) configuration. For (+1, +1) and (−1, −1) configurations, the difference is *μ*_0_. FM contacts 1 and 2 only changes the electrochemical potential in region 1 and 3 respectively and change is equal to separation Δ ≈ *p*_*f*_(*μ*_*up*_ − *μ*_*dn*_). Parameters: *λ* ≈ 100 nm, *M* + *N* ≈ 100, *p*_*c*_ ≈ 0.5, and *p*_*f*_ ≈ 0.9.

We calculate the electrochemical potentials (Eqs (9) and (10)) along the channel by solving Eq. (11) for a fixed charge current *I*_12_ flowing between FM contacts 1 and 2 (see Fig. 4(a)). We assumed point contacts on the channel and the polarization of the FM contacts are set to *p*_*f*_ ≈ 0.9. We look at the case of *p*_*c*_ ≈ 0.5 with spin-flip reflection being the dominant scattering process in the channel (i.e. *r*_*s*1,2_ ≫ *r*, *t*_*s*_). Total number of modes (*M* + *N*) in the channel can be estimated by *k*_*F*_*W*/*π*, where *k*_*F*_ is Fermi wave vector which is ~1.5 nm^−1^ for Bi_2_Se_3_^9^ and *W* is the channel width. We assumed *M* + *N* ≈ 100 and the mean free path 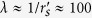 nm, where 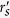 is the spin-flip reflection probability per mode.

### Electrochemical potential profile

Electrochemical potential distributions are shown in Fig. 4(b–e) for FM magnetization configurations (*m*_1_, *m*_2_) = (+1, +1), (+1, −1), (−1, +1), and (−1, −1) respectively. The separation between electrochemical potentials for forward and backward moving states is *μ*^+^ − *μ*^−^ = *qI*_12_/*G*_*B*_^43^. Spin-orbit coupling present in the channel (i.e. *p*_*c*_ ≠ 0) creates a spin potential given by *μ*_*up*_ − *μ*_*dn*_ ≈ *αp*_*c*_(*μ*^+^ − *μ*^−^). The slopes of *μ*^+^ and *μ*^−^ in region 2 are due to Ohmic drop caused by different scattering mechanisms in the channel. No charge current flows in region 1 and 3 in steady state, hence no separation between *μ*^+^ and *μ*^−^ or *μ*_*up*_ and *μ*_*dn*_ and they are flat along the channel.

The results in Fig. 4(b–e) corresponding to the four possible combinations, (*m*_1_, *m*_2_) = (±1, ±1) can be understood simply if we assume the FM contacts to be perfectly polarized (*p*_*f*_ = 1). Since the separation of *μ*_*up*_ and *μ*_*dn*_ changes at each FM contact, one or both of these potentials must change. If *m*_1,2_ = +1 at a particular contact, no down spins can flow in from this contact (assuming *p*_*f*_ = 1), and hence *μ*_*dn*_ does not change, only *μ*_*up*_ changes. Similarly if *m*_1,2_ = −1, only *μ*_*dn*_ changes. Hence the change is equal to the separation (*μ*_*up*_ − *μ*_*dn*_) in the current-carrying region (region 2). With imperfectly polarized contacts this effect is reduced somewhat but the basic effect is evident from the plots in Fig. 4(b–e), which use *p*_*f*_ ≈ 0.9: at each contact most of the change is in the potential corresponding to the majority spin. Note that if high resistance contacts 1 and 2 are NM (i.e. *p_f_ *= 0), the positions of electrochemical potentials in region 1 and 3 will be the average of the *µ^+^* and *µ^−^* at the corresponding contacts. This is different from the electrochemical potential profile due to current injection from low resistance NM contacts, as shown previously in Fig. 1.

### Contact conductance

**Figure 5:**
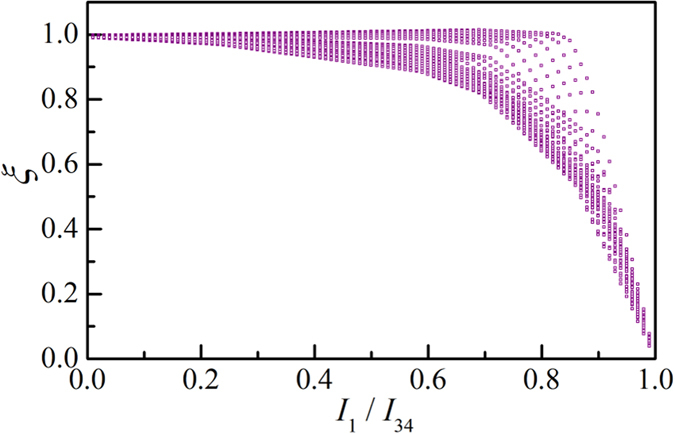
The factor *ξ* as a function of the fraction of the channel current (*I*_1_/*I*_34_) which leaves from the FM contact under short circuit condition. *ξ* becomes significantly less than 1 as the FM contact conductance is such that >70% of the channel current current flows out of the contact under short circuit condition. Each point on the plot corresponds to different values of *p*_*c*_ and *p*_*f*_, where both of them ranges from 0.01 to 1.

We quantify the FM contact resistance by *I*_1_/*I*_34_ which represents the fraction of the channel current that leaves from the FM contact under short circuit condition. *I*_34_ is the supply current between NM contacts 3 and 4 and *I*_1_ is the short circuit current at FM contact 1. We assume that both FM contacts have same contact resistance. *ξ* as a function of *I*_1_/*I*_34_ is shown in Fig. 5 for different values of *p*_*c*_ and *p*_*f*_, both ranging from 0.01 to 1. The factor *ξ* remains close to 1 for high resistance FM contacts (i.e. *I*_1_/*I*_34_ → 0) and becomes significantly less than 1 if the contact resistance is such that >70% of the channel current flows out of the contact under short circuit condition. This effect of contact resistance should be taken into account for efficient device design to read magnetic states through IREE^49^.

Note that Fig. 5 is obtained with *r*, *t*_*s*_ = 0.01*r*_*s*_, assuming that the reflection with spin-flip is the dominant scattering mechanism due to spin-momentum locking. However, if *r*, *t*_*s*_ is comparable to *r*_*s*_ then *ξ* can be different from 1 even for high resistance contacts (i.e. potentiometric limit: *I*_1_/*I*_34_ → 0). In the potentiometric limit, our basic result in Eq. (1) follow from the following relations:


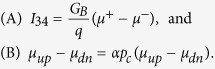


While relation (A) is generally valid^43^, relation (B) is an approximate one. It is exact only if *r* = *t*_*s*_ = 0 so that 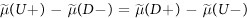. This last relation is satisfied if *U*+, *D*− states are completely decoupled from the *U*−, *D*+ states and behave essentially as independent conductors.

## Summary

We propose a four-terminal spin valve like device on spin-orbit coupling materials, which show three distinct four-terminal resistance due to spin-momentum locking in the channel. Our proposal is based on Onsager reciprocity relation, starting from the prior proposal of multi-terminal potentiometric measurement of charge induced spin voltage on 2D channels with SML which has been confirmed experimentally by several groups. We argue that this effect should not be observed in a two-terminal linear response measurement because of the restrictions of Onsager reciprocity. We support our proposal with detailed numerical calculations based on a generalized spin diffusion equation which uses four electrochemical potentials based on spin polarization and group velocity.

## Additional Information

**How to cite this article**: Sayed, S. *et al*. Multi-Terminal Spin Valve on Channels with Spin-Momentum Locking. *Sci. Rep.* **6**, 35658; doi: 10.1038/srep35658 (2016).

## Supplementary Material

Supplementary Information
