# Supplementary material for: Multi-Terminal Spin Valve on Channels with Spin-Momentum Locking
Source: Sci Rep. 2016 Oct 21;6:35658. doi: 10.1038/srep35658 (PMC5073362; doi:10.1038/srep35658)
Supplement: Supplementary Information [file srep35658-s1.pdf]

Multi-Terminal Spin Valve on Channels with Spin-Momentum Locking
Supplementary Information

Shehrin Sayed, Seokmin Hong, and Supriyo Datta
School of Electrical and Computer Engineering,
Purdue University, West Lafayette, IN 47907, USA

I. NUMERICAL SETUP

This section provides the numerical method used to solve Eq. (11) *in the main manuscript.*

We have discretized Eqs. (11) and (12) in the main manuscript using finite difference method considering the up-winding [1] scheme:

$$\begin{aligned}
\frac{d}{dx}\tilde{\mu}(U+) &\equiv \frac{\tilde{\mu}(U+)_n - \tilde{\mu}(U+)_{n-1}}{a}, \\
\frac{d}{dx}\tilde{\mu}(D+) &\equiv \frac{\tilde{\mu}(D+)_n - \tilde{\mu}(D+)_{n-1}}{a}, \\
\frac{d}{dx}\tilde{\mu}(U-) &\equiv \frac{\tilde{\mu}(U-)_{n+1} - \tilde{\mu}(U-)_n}{a}, \\
\text{and, } \frac{d}{dx}\tilde{\mu}(D-) &\equiv \frac{\tilde{\mu}(D-)_{n+1} - \tilde{\mu}(D-)_n}{a},
\end{aligned} \tag{I.1}$$

which results into the following form:

$$[\beta_L]\{\varphi\}_{n-1} + [\alpha]\{\varphi\}_n + [\beta_R]\{\varphi\}_{n+1} = -q\,[g_C]_i\,\{V_i\}_n \tag{I.2}$$

for $n = 1$ to K. Here, K is the number of lattice points, a is the distance between two lattice points, q is the electron charge, $\{V_i\}$ is the terminal voltage in up-down basis at i^{th} contact, $[g_C]_i$ is the normalized contact conductance matrix of ith contact. Note that $\{\varphi\}_0$ and $\{\varphi\}_{K+1}$ are boundary conditions at left and right ends of the channel respectively. The vectors and matrices are given by

$$\{\varphi\}_n = \begin{Bmatrix} \tilde{\mu}(U+) \\ \tilde{\mu}(D-) \\ \tilde{\mu}(U-) \\ \tilde{\mu}(D+) \end{Bmatrix}_n, \quad \{V_i\}_n = \begin{Bmatrix} v_u \\ v_d \\ v_u \\ v_d \end{Bmatrix}_i, \quad [g_C]_i = \begin{bmatrix} g'_u & 0 & 0 & 0 \\ 0 & g'_d & 0 & 0 \\ 0 & 0 & g'_u & 0 \\ 0 & 0 & 0 & g'_d \end{bmatrix}_i,$$

$$[\alpha] = \begin{bmatrix} -u'_1 - 1 - g'_u & r'_{s1} & r'\frac{N}{M} & t'_s\frac{N}{M} \\ r'_{s1} & -u'_1 - 1 - g'_d & t'_s\frac{N}{M} & r'\frac{N}{M} \\ r' & t'_s & -u'_2 - 1 - g'_u & r'_{s2} \\ t'_s & r' & r'_{s2} & -u'_2 - 1 - g'_d \end{bmatrix},$$

$$[\beta_L] = \begin{bmatrix} 1 & 0 & 0 & 0 \\ 0 & 0 & 0 & 0 \\ 0 & 0 & 0 & 0 \\ 0 & 0 & 0 & 1 \end{bmatrix}, \text{ and } [\beta_R] = \begin{bmatrix} 0 & 0 & 0 & 0 \\ 0 & 1 & 0 & 0 \\ 0 & 0 & 1 & 0 \\ 0 & 0 & 0 & 0 \end{bmatrix},$$

with $g'_u = (a\,g_u)/(q^2/h)$, $g'_d = (a\,g_d)/(q^2/h)$, $u'_1 = r'_{s1} + r'\frac{N}{M} + t'_s\frac{N}{M}$, $u'_2 = r'_{s2} + r' + t'_s$, $t'_s = (a\,t_s)/N$, $r' = (a\,r)/N$, $r'_{s1} = (a\,r_{s1})/M$, and $r'_{s2} = (a\,r_{s2})/N$. We use Eq. (I.2) to construct multi-contact based structures as shown in Fig. S1.

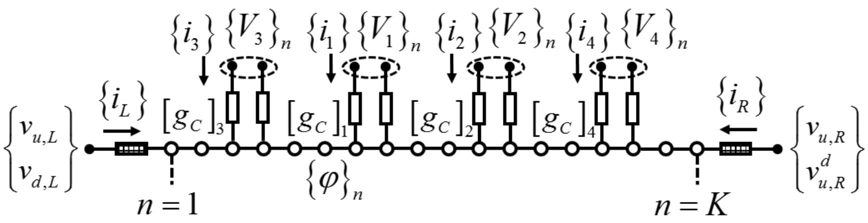

FIG. S1. Numerical setup using Eq. (I.2) for multi-contact based structures on channels with spin-momentum locking. The terminals with the subscript L and R are the left and right boundaries of the channel. i^{th} contact ($i = 1, 2, 3,$ and 4) will be a NM contact if $g_u = g_d$ or a FM contact if $g_u \neq g_d$.

A. Contact Currents

We multiply both sides of Eq. (I.2) with $[m] = diag\left(\left[\begin{array}{cccc} M & M & N & N \end{array}\right]\right)$ and sum over all lattice points as

$$\sum_{n=1}^{K}\left([m]\left[\beta_L\right]\{\varphi\}_{n-1}+[m]\left[\alpha\right]\{\varphi\}_{n}+[m]\left[\beta_R\right]\{\varphi\}_{n+1}+q[m]\left[g_C\right]_i\{V_i\}_n\right)=0,$$

which gives

$$\begin{aligned} &a\sum_{n=1}^{K}[S]\{\varphi\}_n+\sum_{n=1}^{K}[m]\left[g_C\right]_i\left(q\{V_i\}_n-\{\varphi\}_n\right)\\ &+[m]\left(\left[\beta_L\right]\{\varphi\}_0-\left[\beta_R\right]\{\varphi\}_1\right)+[m]\left(\left[\beta_R\right]\{\varphi\}_{K+1}-\left[\beta_L\right]\{\varphi\}_K\right)=0, \end{aligned} \tag{I.3}$$

representing the sum of all up and down spin polarized currents flowing in/out of the channel. The scattering matrix is given by

$$[S]=\left[\begin{array}{cccc} -u_1 & r_{s1} & r & t_s \\ r_{s1} & -u_1 & t_s & r \\ r & t_s & -u_2 & r_{s2} \\ t_s & r & r_{s2} & -u_2 \end{array}\right] \text{ with } u_1=r_{s1}+r+t_s, \text{ and } u_2=r_{s2}+r+t_s.$$

The first term which is related to the scattering processes in the channel vanishes for charge current (i.e. $\left[\begin{array}{cccc} 1 & 1 & 1 & 1 \end{array}\right]\sum_{n=1}^{N}\left([S]\{\varphi\}_n\right)=0$) satisfying the charge current conservation law. This term is non-zero for spin current and captures the spin loss in the channel due to spin relaxation.

The second term is the up and down spin polarized currents flowing in the channel from the ith external contact, which is the Eq. (12) of the main manuscript. In the discrete lattice space, the total current is the sum over all the lattice points where the contact is connected, given by

$$\left\{\begin{array}{c} i_u \\ i_d \end{array}\right\}_i = a(M+N)\sum_j\left\{\begin{array}{c} g_u\left(v_{u,i}-\dfrac{M\,\tilde{\mu}_j(U+)+N\,\tilde{\mu}_j(U-)}{q\,(M+N)}\right) \\ g_d\left(v_{d,i}-\dfrac{M\,\tilde{\mu}_j(D-)+N\,\tilde{\mu}_j(D+)}{q\,(M+N)}\right) \end{array}\right\}. \tag{I.4}$$

Note that the contact can be ferromagnetic if $g_u \neq g_d$ or non-magnetic if $g_u = g_d$.

The third and fourth terms are the up/down spin polarized currents at the left and right ends of the channel respectively, which gives Eq. (13) in the main manuscript. Boundary conditions at left and right ends are $\{\varphi\}_0 \equiv \left\{\begin{array}{cccc} v_{u,L} & v_{d,L} & v_{u,L} & v_{d,L} \end{array}\right\}^T$ and $\{\varphi\}_{K+1} \equiv \left\{\begin{array}{cccc} v_{u,R} & v_{d,R} & v_{u,R} & v_{d,R} \end{array}\right\}^T$ respectively, which

yields

$$\left\{\begin{array}{c} i_u \\ i_d \end{array}\right\}_L = \frac{q^2}{h}\left\{\begin{array}{c} Mv_{u,L} - N\dfrac{\tilde{\mu}_1\left(U-\right)}{q} \\ Nv_{d,L} - M\dfrac{\tilde{\mu}_1\left(D-\right)}{q} \end{array}\right\},$$

$$\text{and} \left\{\begin{array}{c} i_u \\ i_d \end{array}\right\}_R = \frac{q^2}{h}\left\{\begin{array}{c} Nv_{u,R} - M\dfrac{\tilde{\mu}_K\left(U+\right)}{q} \\ Mv_{d,R} - N\dfrac{\tilde{\mu}_K\left(D+\right)}{q} \end{array}\right\}. \tag{I.5}$$

We can convert the vectors and matrices from up-down to charge-spin basis by multiplying the following matrix:

$$[\tau] = \begin{bmatrix} 1 & 1 \\ 1 & -1 \end{bmatrix}. \tag{I.6}$$

B. Boundary Conditions

In all our simulations, we have applied the open circuit boundary condition at the charge terminal and short circuit boundary condition at the spin terminal for the contacts at the left and the right ends of the channel:

$$\left\{\begin{array}{c} i_c \\ v_s \end{array}\right\}_L = \left\{\begin{array}{c} i_c \\ v_s \end{array}\right\}_R = \left\{\begin{array}{c} 0 \\ 0 \end{array}\right\}, \tag{I.7}$$

where i_c and v_s are terminal charge current and spin voltage respectively. If i^{th} terminal is used to measure an open circuit charge voltage, then the boundary condition at the terminal is given by:

$$\left\{\begin{array}{c} i_c \\ v_s \end{array}\right\}_i = \left\{\begin{array}{c} 0 \\ 0 \end{array}\right\}. \tag{I.8}$$

On the other hand, if the ith terminal is used for supply, then the boundary condition at the terminal is given by:

$$\left\{\begin{array}{c} i_c \\ v_s \end{array}\right\}_i = \left\{\begin{array}{c} I_0 \\ 0 \end{array}\right\} \text{ or } \left\{\begin{array}{c} v_c \\ v_s \end{array}\right\}_i = \left\{\begin{array}{c} V_0 \\ 0 \end{array}\right\}, \tag{I.9}$$

where v_c is the terminal charge voltage, I_0 is the external current source, and V_0 is the external voltage source.

[1] S. V. Patankar, Numerical Heat Transfer and Fluid Flow, (Taylor and Francis, ISBN 978-0-89116-522-4, 1980).
